# Supplementary material for: Multi-channel portable odor delivery device for self-administered and rapid smell testing
Source: Commun Eng. 2024 Oct 11;3:141. doi: 10.1038/s44172-024-00286-1 (PMC11470141; doi:10.1038/s44172-024-00286-1)
Supplement: Supplementary file 2 — Supplementary Information [file 44172_2024_286_MOESM2_ESM.pdf]

Multi-channel portable odor delivery device for self-administered and rapid smell testing

Richard Hopper[1,2*], Daniel Popa[2*], Emanuela Maggioni[1,3*], Devarsh Patel[1], Marianna Obrist[3], Basile Nicolas Landis[4], Julien Wen Hsieh[4], Florin Udrea[2]

[1]OWidgets Ltd. - OW Smell Made Digital, London, E1 1DU, UK.
[2]Department of Engineering, University of Cambridge, Cambridge, CB3 0FA, UK.
[3]Department of Computer Science, University College London, London, NW1 2AE, UK.
[4]Rhinology-Olfactory Unit, Department of Otorhinolaryngology-Head and Neck Surgery, Geneva University Hospitals, Geneva, 1205, Switzerland.

*Corresponding author(s). E-mail(s): richard@owidgets.co.uk; dp387@cam.ac.uk; emanuela@owidgets.co.uk;
Contributing authors: devarsh@owidgets.co.uk; m.obrist@ucl.ac.uk; basile.landis@hcuge.ch; hsiehjulien@gmail.com; fu@eng.cam.ac.uk;

Supplementary Figures

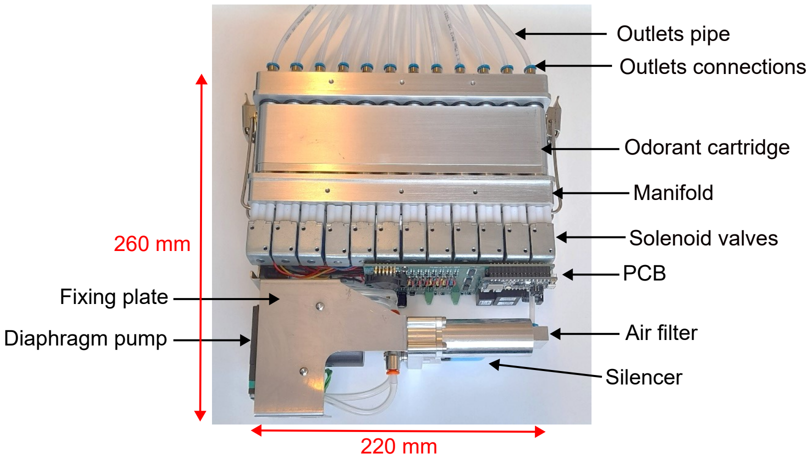

Supplementary Figure 1: | **Device internals.** Image of the internals of the odor delivery device with the case removed showing the main system components, including the diaphragm pump for airflow generation, air filter, solenoid valves, odorant cartridge, outlet pipes and printed circuit board (PCB) for system control.

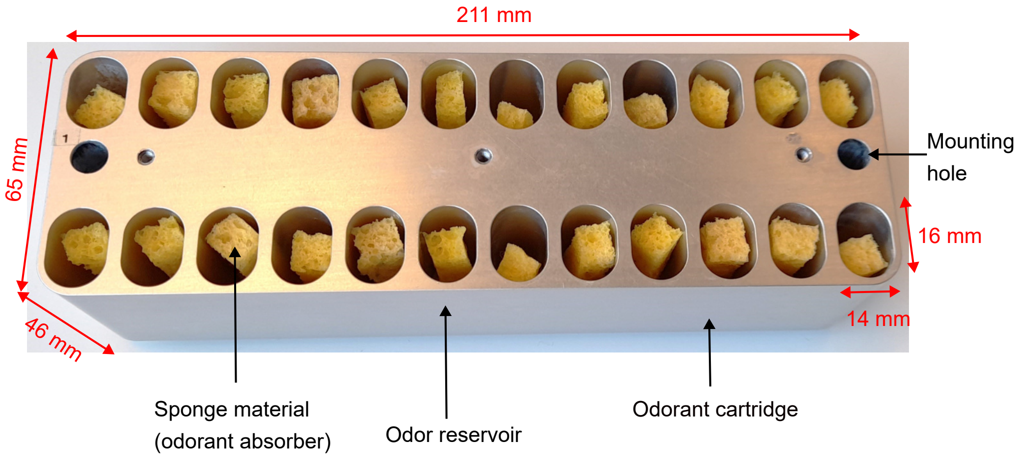

Supplementary Figure 2: | **Odorant cartridge.** Image of the metal odorant cartridge of the odor delivery device showing the 24 odor reservoirs containing the sponge absorber material saturated with the odorant.

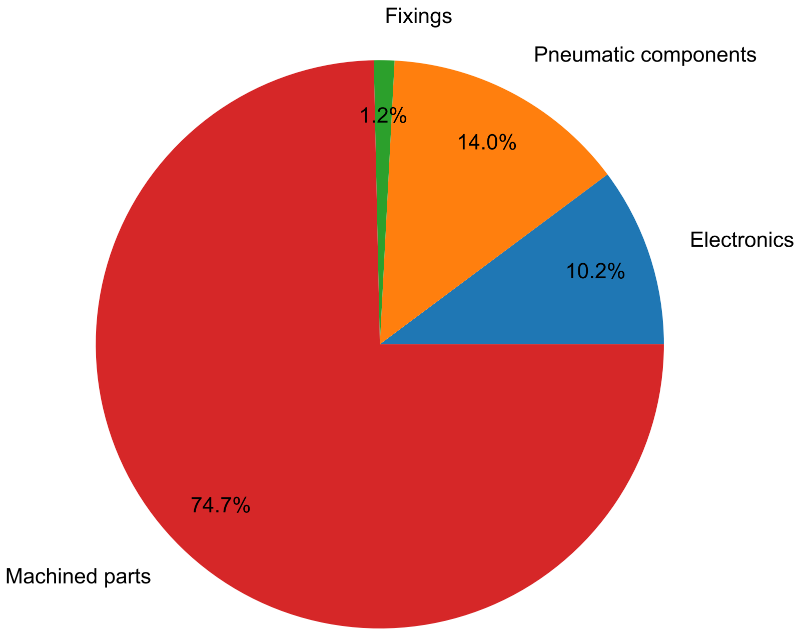

Supplementary Figure 3: | **Cost breakdown.** A pie chart showing the relative cost of the different system components making up the odor delivery device. The cost of the custom machined parts dominates. The total cost for parts was $2,140.

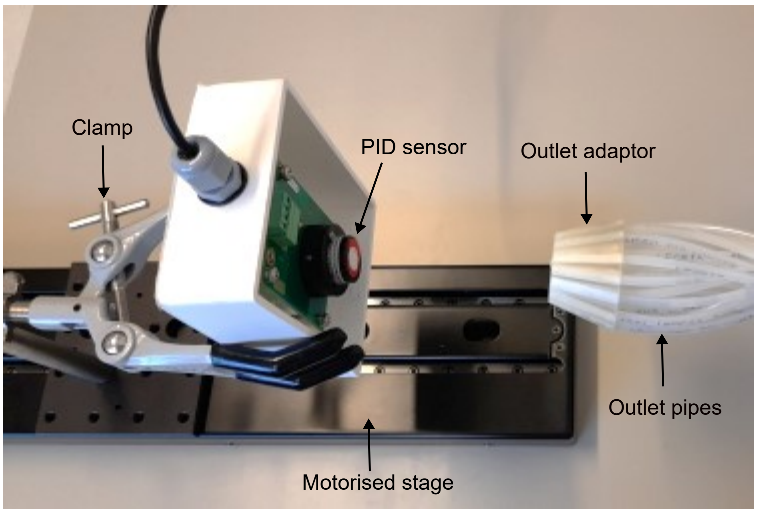

Supplementary Figure 4: | **Odor mapping test setup.** Experimental setup used for mapping the odor distribution. A motorised stage was used to position the photoionization detector (PID) at different distances from the outlet of the odor delivery device.
