# Supplementary material for: Multi-channel portable odor delivery device for self-administered and rapid smell testing
Source: Commun Eng. 2024 Oct 11;3:141. doi: 10.1038/s44172-024-00286-1 (PMC11470141; doi:10.1038/s44172-024-00286-1)
Supplement: Supplementary file 3 — Description of Additional Supplementary Files [file 44172_2024_286_MOESM3_ESM.pdf]

Description of Additional Supplementary Files

File name: Supplementary Data 1

Description: Cost breakdown. Data showing the cost of the different system components making up the odor delivery device.

File name: Supplementary Data 2

Description: Source Data for Figures 2 and 3.
